# Molecular typing and antibiotic resistance patterns among clinical isolates of *Acinetobacter baumannii* recovered from burn patients in Tehran, Iran

**DOI:** 10.3389/fmicb.2022.994303

**Published:** 2022-10-21

**Authors:** Abbas Maleki, Vahab Hassan Kaviar, Maryam Koupaei, Mohammad Hossein Haddadi, Behrooz Sadeghi Kalani, Hassan Valadbeigi, Somayeh Karamolahi, Nazanin Omidi, Marziyeh Hashemian, Nourkhoda Sadeghifard, Jasem Mohamadi, Mohsen Heidary, Saeed Khoshnood

**Affiliations:** ^1^Clinical Microbiology Research Center, Ilam University of Medical Sciences, Ilam, Iran; ^2^Department of Microbiology and Immunology, School of Medicine, Kashan University of Medical Sciences, Kashan, Iran; ^3^Department of Microbiology, School of Medicine, Ilam University of Medical Sciences, Ilam, Iran; ^4^Department of Pediatrics, School of Medicine, Imam Khomeini Hospital, Ilam University of Medical Sciences, Ilam, Iran; ^5^Department of Laboratory Sciences, School of Paramedical Sciences, Sabzevar University of Medical Sciences, Sabzevar, Iran; ^6^Cellular and Molecular Research Center, Sabzevar University of Medical Sciences, Sabzevar, Iran

**Keywords:** *Acinetobacter baumannii*, extensively drug-resistant, multidrug-resistant, multilocus sequence typing, tigecycline

## Abstract

*Acinetobacter baumannii* (*A. baumannii*) is now considered a highly resistant pathogen to various types of antibiotics. Therefore, tracking the source of its prevalence and continuous control is crucial. This study aimed to determine antibiotic resistance and perform various molecular typing methods on clinical isolates of *A. baumannii* isolated from hospitalized burn patients in Shahid Motahari Burn Hospital, Tehran, Iran. Hospital isolates were confirmed by phenotypic and molecular methods. Then the sensitivity to different antibiotics was determined using the minimum inhibitory concentration (MIC) method. In order to perform molecular typing, three-locus dual assay multiplex polymerase chain reaction (PCR), multiple-locus variable-number tandem repeat analysis (MLVA), and multilocus sequence typing (MLST) methods were used. Among the 60 isolates collected, the frequencies of multidrug-resistant (MDR) and extensively drug-resistant (XDR) isolates were 90 and 10%, respectively. The most effective antibiotics were colistin with 100% and tigecycline with 83.33% sensitivity. Isolates were 100% resistant to piperacillin/tazobactam and cephalosporins, and 68.3% were resistant to carbapenem. The results of multiplex PCR showed five groups that international clone I (IC I) and IC II were the most common. The MLVA method identified 34 MLVA types (MTs), 5 clusters, and 25 singletons. Multilocus sequence typing results for tigecycline-resistant isolates showed seven different sequence types (STs). Increasing antibiotic resistance in *A. baumannii* isolates requires careful management to control and prevent the occurrence of the pre-antibiotic era. The results of this study confirm that the population structure of *A. baumannii* isolates has a high diversity. More extensive studies are needed in Iran to better understand the epidemiology of *A. baumannii*.

## Introduction

Non-fermenting gram-negative bacteria bacterium, *Acinetobacter baumannii*, is a nosocomial pathogen in hospitals (Gholami et al., 2017; Sato et al., 2021). It can be responsible for a vast range of nosocomial severe infections such as ventilator-associated pneumonia (VAP), bloodstream infections, skin, and soft tissue infections, wound infections, urinary tract infections (UTI), and meningitis (Ayoub Moubareck and Hammoudi, 2020). Owing to high resistance to commonly used antibiotics, more than 80% of *Acinetobacter* species are regarded as multidrug-resistant (MDR). In addition, because of the inability to treat the infections caused by this bacterium, it has also increased the rate of mortality in patients, imposing a tremendous burden on health care (Khoshnood et al., 2017; Shi et al., 2020). Lately, a dramatic rise has been seen in the number of extensively drug-resistant (XDR) and pandrug-resistant (PDR) *A. baumannii* isolates as well (Mirnejad et al., 2018; Teerawattanapong et al., 2018).

Due to its considerable threat to public health, the World Health Organization (WHO) has introduced *A. baumannii* as a crucial priority pathogen toward which the production of new antibiotics is essential (Jiang et al., 2021). Carbapenem antibiotics, such as meropenem and imipenem, are the most effective antibacterial agents for *A. baumannii* isolates. The rapid spread of carbapenem-resistant MDR *A. baumannii* (CRAB) has led to the use of other antibiotics. For treatment of clinical MDR bacterial infections, the third-generation tetracycline antibiotic, tigecycline, is used as one of the final-resort antibiotics (Chen et al., 2020). Protein synthesis is adversely affected by hindering aminoacyl-tRNA entry into the aminoacyl (A) site of the 30S ribosome in prokaryotic translation (Ahmad et al., 2019). Tigecycline is prescribed to treat severe infections such as infectious diseases caused by MDR *A. baumannii* isolates (Owrang et al., 2018).

Nevertheless, resistance to tigecycline and colistin in *A. baumannii* has increased and is very concerning (Dortet et al., 2018; Hua et al., 2021). Percentages of tigecycline non-susceptibility rates against *A. baumannii* in Asia have been reported to be 14.2 to 57.6% (Xu et al., 2020). Follow-up research on the susceptibility profile of these two antibiotics is necessary for public health worldwide. The epidemiology analysis of *A. baumannii* is needed to comprehend its genetic variation and raise awareness about treating and controlling this bacterial infection (Bian et al., 2021). Several effective typing methods have been employed to determine the genetic diversity, strains’ relatedness, and the epidemiology of *A. baumannii*. One of these typing methods consists of two multiplex polymerase chain reactions (PCRs) to amplify alleles of the gene encoding outer membrane protein A (*ompA*), the gene encoding part of a pilus assembly system required for biofilm formation (*csuE*), and the gene encoding the intrinsic carbapenemase gene of *A. baumannii* (*bla*_OXA_-51-like) that can be utilized to recognize international clones (ICs) I, II, and III (Turton et al., 2007). This method performs a preparatory typing approach to rapidly investigate the hospital epidemiology (Giannouli et al., 2009). Other typing methods, multiple-locus variable-number tandem repeat analysis (MLVA), and multilocus sequence typing (MLST), can be mentioned as well (Farshadzadeh et al., 2015). An interesting source of genetic polymorphism is provided by tandemly repeated sequences, known as variable number of tandem repeats (VNTRs), whose number of repetitions varies at different rates depending on the different loci and even alleles. Molecular typing based on the analysis of repeat copy number at multiple VNTR loci, known as MLVA, is a genotyping method that is being used for strain comparison, and can also provide insights into population structure (Dahyot et al., 2018). This method has some benefits such as high resolution, data portability, and intra-laboratory reproducibility (Lindstedt et al., 2013). Multilocus sequence typing described as a nucleotide sequence-based method providing an allelic profile or a sequence type (ST) for each isolate, is now extremely considered a supplementary tool for global epidemiological research that offers possibilities for the identification of epidemics and virulence of *A. baumannii* clones and the monitoring of their spread at both the national and international levels (Khuntayaporn et al., 2021). Both approaches (MLVA and MLST) allow for identification of clonal lineages and the investigation of genetic diversity of the clinical *A. baumannii* clones (Hauck et al., 2012). The main aim of the current study was to evaluate the antibiotic resistance and molecular typing of clinical isolates of *A. baumannii* collected from hospitalized burn patients in Shahid Motahari Burn Hospital, Tehran, Iran.

## Materials and methods

### Bacterial isolates and identification

This cross-sectional study received ethical approval from the local ethics committee of Ilam University of Medical Sciences (IR.MEDILAM.REC.1400.135). All patients provided written informed consent. From the beginning of October 2020 to the end of July 2021, 60 non-duplicate *A. baumannii* isolates were obtained from 111 various clinical specimens (catheter, pleural fluid, tracheal aspirates, blood, cerebrospinal fluid (CSF), and urine) taken from hospitalized patients in Shahid Motahari burn hospital in Tehran, Iran. Isolates were streaked on blood agar (blood agar base by Merck Chemicals, Darmstadt, supplemented with 5% sheep blood) and MacConkey agar (Merck, Darmstadt, Germany) and incubated aerobically at 37°C for 24 h. Primary identification *A. baumannii* isolates was based on the colony morphology and Gram staining reaction. Standard biochemical tests such as citrate, oxidase, catalase, triple sugar iron agar, methyl red, Voges Prausker, urease test, oxidation and fermentation of sugars, and indole production were used to identify the *A. baumannii* isolates (Forbes, 2007). Identification of *A. baumannii* was confirmed by PCR detection of the *bla*_OXA_-51-like gene according to Turton et al. (Turton et al., 2006) and *gyrB* gene based on the method of Higgins et al. (Higgins et al., 2007). The *A. baumannii* ATCC 19606 was used as a positive control.

### Susceptibility testing

Minimum inhibitory concentrations (MICs) were determined using the VITEK 2 system (bioMérieux, Marcy l’Etoile, France) for the following seven antimicrobial categories: β-lactam/β-lactamase inhibitors combinations (Piperacillin/tazobactam PIP/TAZ), extended-spectrum cephalosporins (ceftazidime (CAZ), cefepime (CEP)), carbapenems (imipenem (IMI), meropenem (MER), Lipopeptide (colistin, COL), aminoglycosides (gentamicin(GEN), tobramycin (TOB), tetracyclines (tigecycline (TGC), tetracycline (TET), and fluoroquinolones (ciprofloxacin (CIP). The results were evaluated according to the Clinical and Laboratory Standards Institute (CLSI) criteria (Wayne, 2020), except for tigecycline, in which case the results were evaluated according to the criteria suggested by Jones et al. (2007); MIC ≥8 μg/ml, as the resistant breakpoint. Multidrug resistance profile as defined by the isolate being non-susceptible to at least one agent in ≥3 antimicrobial categories. Isolates of *A. baumannii* with resistance to at least one agent in all but two or fewer antimicrobial categories were considered XDR (Magiorakos et al., 2012). Reference strains *Escherichia coli* ATCC 25922 and *Pseudomonas aeruginosa* ATCC 27853 were used as quality controls.

### Molecular typing methods

#### Multiplex PCR

The sequence group (SG) or IC type of the isolates were determined by using two multiplex PCR assays designed to selectively amplify *ompA*, *csuE* and *bla*_OXA_-51-like genes (Turton et al., 2007). The PCR tests were carried out in a final volume of 25-μL containing 3 μl of extracted DNA, 10 pmol of each primer and 1.5 U of Taq DNA polymerase in 1 × PCR buffer containing 1.5 mM MgCl_2_ (Qiagen, Crawley, United Kingdom) and 200 μM each dNTP. PCR mixtures were subjected to the following thermal cycling: 3 min at 94°C, followed by 30 cycles with denaturation at 94°C for 45 s, annealing at 46–65°C for 45 s, extension at 72°C for 1 min, and a final extension at 72°C for 5 min (Turton et al., 2007). Identification of a strain as a member of Group 1 (G1) or Group 2 (G2) required the amplification of all fragments in the corresponding multiplex PCR and an absence of any amplification by the other multiplex PCR. Group 3 (G3) isolates were defined by the amplification of only the *ompA* fragment in the Group 2 PCR and the amplification of only the *csuE* and *bla*_OXA-51_-like fragments in the Group 1 PCR (For other groups, see Supplementary File 1). According to the new combination of amplified products, and isolates pertained to the novel variant of the PCR-based group. Isolates were grouped in G1 (IC II), G2 (IC I), and G3 (IC III). Other isolates that did not belong to these types were reported as the IC variants.

#### Multiple loci VNTR analysis

Isolates were genotyped using the MLVA-8 scheme method developed by Pourcel et al. (2011). The MLVA-8 scheme profiles in each isolate were identified by the number of repeats estimated at each VNTR locus. The bacterial genomic DNA was extracted using the boiling method (Gomaa et al., 2017) and its concentration was assessed using a NanoDrop ND-1000 spectrophotometer (NanoDrop Technologies, Wilmington, DE, USA). Oligonucleotide primers targeting the flanking regions of the S-repeat VNTRs (Abaum3468, Abaum0845, Abaum0826, and Abaum2396) and L-repeat VNTRs (Abaum-3,530, Abaum3002, Abaum2240, and Abaum1988) were used for the amplification of *A. baumannii* genomic DNA. Small (S)-repeat VNTRs were the loci with a repeat unit size of up to 9 bp, whereas L (Large)-repeat VNTR loci were the loci with a repeat unit size of above 9 bp. In the following, PCRs were performed in a 25 μl final volume containing 20 pM of each primer, 200 μM of each deoxynucleoside triphosphate (dNTPs), 1.5 mM MgCl_2_, 3 ng genomic DNA, ×1′ reaction buffer, and1Uof Taq DNA polymerase. Amplification cycles included an initial 94°C denaturation for 5 min, followed by 36 cycles of 94°C for 30, 30 s of annealing at 50°C for Abaum0826 and Abaum0845 and at 55°C for the remainder loci, elongation at 72°C for 30 s, and a final elongation at 72°C for 10 min. Then, 5 μl of each PCR amplicon solution were analyzed on 3% agarose gel (SinaClon BioScience, Iran) by electrophoresis in 1× Tris–borate-EDTA (TBE) buffer. Size markers included a 50-bp DNA standards (Thermo Scientific) and 100-bp (SinaClon Bioscience), for S- and L-VNTRs, respectively. The size of the amplicon was determined using GeneTools v.3.08 automatic image analysis software (Syngene, Cambridge, United Kingdom). The number of repeats in VNTR alleles for isolates was estimated by subtracting the flanking region size from the amplicon size and then dividing by the repeat unit length. Primers, PCR conditions and characteristics of VNTRs used in this study are shown in Supplementary File 2. A cut-off value of 90% similarity was applied to define clusters, and MLVA type (MT) was determined using a 100% similarity cut-off, as previously described (Nhu et al., 2014).

### Multilocus sequence typing

Seven housekeeping genes (*gltA*, *gyrB*, *gdhB*, *recA*, *cpn60*, *gpi*, and *rpoD*) were amplified and sequenced to determine the genotype. DNA sequence variations and STs were analyzed using the MLST database for *A. baumannii*.^1^ MLST was performed using the Oxford scheme as previously described (Bartual et al., 2005). This typing method was carried out for isolates with resistance to tigecycline (MICs ≥8 μg/ml).

#### Statistical analysis

Statistical analysis was performed using the “IBM SPSS statistics 22” software (IBM analytics; United States). Statistical significance of variables determined by chi-square and Fisher’s exact tests. The results are presented as descriptive statistics in terms of relative frequency. A *p*-value of ≤0.05 was considered statistically significant.

## Results

### Studied population and bacterial isolates

*A. baumannii* isolates were identified by various tests that included: Gram-negative coccobacilli, oxidase negative, catalase positive, non-fermentative (oxidative), urease negative, non-fastidious, gas negative, citrate positive, H_2_S negative, indole negative, methyl red positive, and Voges-Proskauer negative. In the present study, 60 non-duplicative *A. baumannii* isolates were collected from 23 (38.3%) females and 37 (61.6%) males with the mean age of 41.4 ± 9 (range 4–75) years, and with the maximum number of cases in the age group of 41 to 60 years (*n* = 28). These isolates were obtained from different clinical specimens, including catheter (23, 38.3%) pleural fluid (7, 11.6%), tracheal aspirates (18, 30%), blood (6, 10%), CSF (3, 5%), and urine (3, 5%). All the 60 isolates originated from the following wards: (5, 8.3%) from infectious diseases, as well as (2, 3.3%) from pediatrics, (11, 18.3%) from surgery, (3, 5%) from urology, (4, 6.6%) from general and (35, 58.3%) from intensive care unit (ICU).

### Antibiotic susceptibility testing

The results showed that colistin and tigecycline with the sensitivity rates of 100% (MICs range from 0.125 to 1 μg/ml) and 83.33% (MICs range from 0.25 to 4 μg/ml) were the most active antibiotics, respectively, while PIP/TAZ and cephalosporins with 100% resistance had no effect on *A. baumannii* isolates (Table 1). The MIC range of tigecycline in all resistant *A. baumannii* isolates were from 8 to 64 μg/ml. Besides, resistance to other antibiotics was ≥65% (ciprofloxacin = 86.66%, imipenem = 65%, meropenem = 68.33%, gentamicin = 86.66%, tobramycin: 68.33% and tetracycline: 70%). According to antibiotic susceptibility testing, among 60 *A. baumannii* isolates screened, 54 (90%) and 6 (10%) were MDR and XDR, respectively. Also, 41 isolates (68.33%) were CRAB with the MICs range from 8 to ≥256 μg/ml.

**Table 1:** Antibiotic susceptibility pattern of 60 *A. baumannii* isolates using MIC determination against 11 different antibiotics.

Susceptibility pattern	Antibiotic resistance No (%)											
		TET	PIP/TAZ	GEN	TOB	CIP	IMI	MER	CTZ	CFP	TGC	COL
	R	42 (70.0)	60 (100)	52 (86.66)	41 (68.33)	52 (86.66)	39 (65)	41 (68.33)	60 (100)	60 (100)	10 (16.66)	0 (0.0)
	I	0 (0.0)	0 (0.0)	0 (0.0)	2 (3.33)	0 (0.0)	0 (0.0)	0 (0.0)	0 (0.0)	0 (0.0)	0 (0.0)	0 0.0)
	S	18 (30.0)	0 (0.0)	8 (13.33)	17 (28.33)	8 (13.33)	21 (35)	19 (0.0)	0 (0.0)	0 (0.0)	50 (83.33)	60 (100)
Interpretive categories and MIC breakpoints (μg/ml)	R	≥16	≥128/4	≥16	≥16	≥4	≥8	≥8	≥32	≥32	-	≥4
	I	8	32/4–64/4	8	8	2	4	4	16	16	-	≤2
	S	≤4	≤16/4	≤4	≤4	≤1	≤2	≤2	≤8	<8		-
MIC range (μg/ml)		2 to ≥256	128 to ≥256	1 to ≥256	2 to ≥256	0.5 to ≥128	2 to ≥128	2 to ≥256	32 to ≥256	32 to ≥256	0.25 to 64	0.125 to 1
IC No (%)	IC I (12)	10 (83.3)	12 (100)	11 (91.6)	9 (75)	12 (100)	7 (58.3)	8 (66.6)	12 (100)	12 (100)	3 (25)	0 (0.0)
	IC II (33)	27 (81.8)	33 (100)	26 (66.6)	19 (57.5)	27 (81.8)	22 (66.6)	25 (75.7)	33 (100)	33 (100)	5 (15.1)	0 (0.0)
	IC variant (15)	5 (33.3)	15 (100)	15 (100)	13 (86.6)	13 (86.6)	10 (66.6)	8 (53.3)	15 (100)	15 (100)	2 (13.1)	(0.0)
	Total (60)	42	60	52	41	52	39	41	60	60	10	0

CTZ, ceftazidime; CFP, cefepime; PIP/TAZ, piperacillin/tazobactam; TGC, tigecycline; COL, colistin; CIP, ciprofloxacin; IMI, imipenem; MEM, meropenem; GEN, gentamicin; TOB, tobramycin; TET, tetracyclin; R, resistant; I, intermediate; S, sensitive; IC, international clone.

### Molecular typing

**Figure 1:**
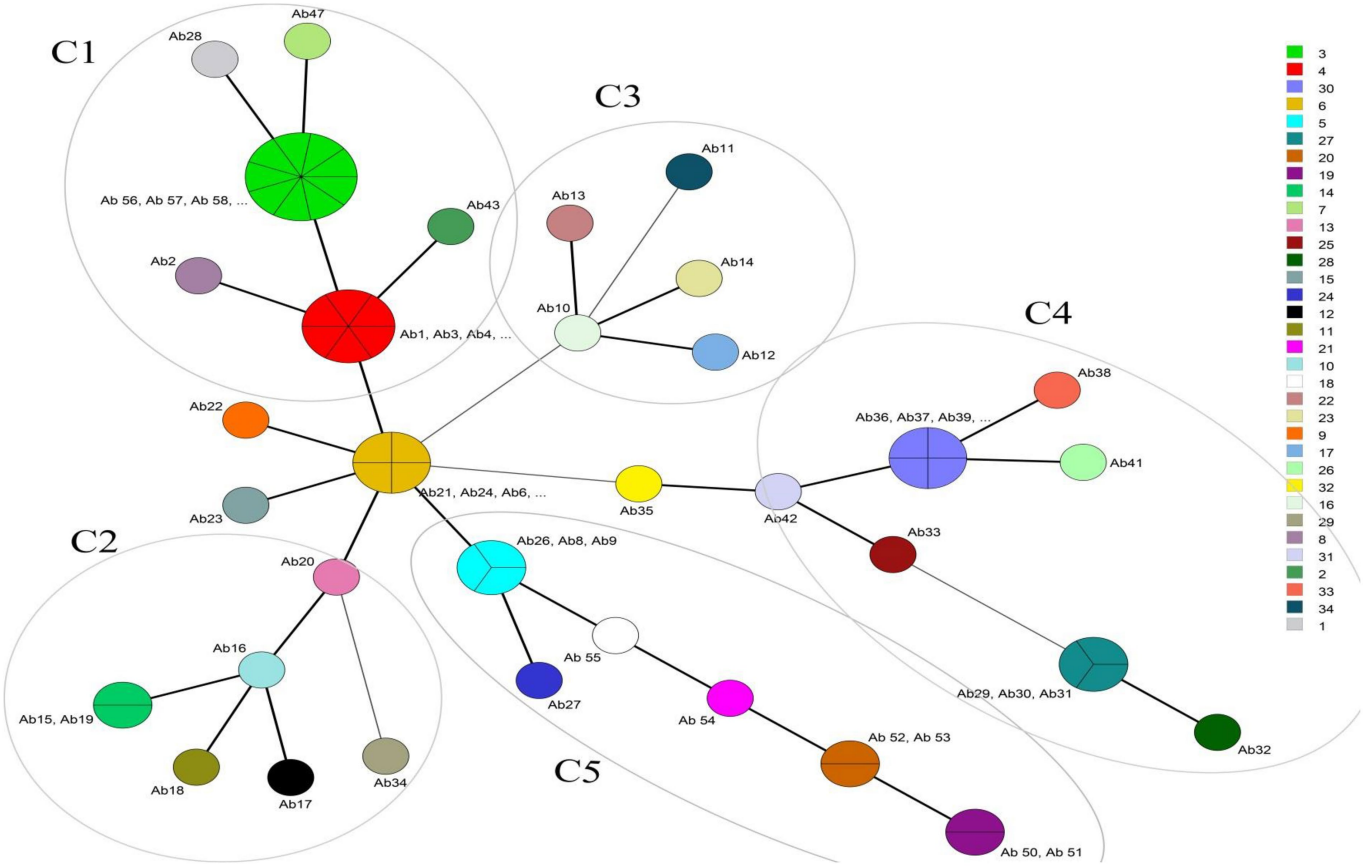
Minimum spanning tree of 60 *A. baumannii* isolates detected for MLVA types (MTs). The right column shows different MTs. Each circle represents isolate number (Ab). The size of each circle indicates the number of isolates within these MTs. Strains that fall into the same MT type are shown with the same color. For example Ab1, Ab3, Ab4, Ab5, Ab44, and Ab45 are located in MT4 because they have the same VNTR numbers (6 7 5 5 9 12 8 12). The different clusters (C1-C5) are annotated. The most isolates were in cluster 1(C1, *n* = 19) and cluster 4 (C4, *n* = 12), respectively. The highest diversity of MTs is in cluster 4. Thick and short lines connecting two types denote types differing in a single locus; thin, longer lines connect double-locus variants; and dashed lines indicate the most likely connection between two types differing in more than two loci.

**Figure 2:**
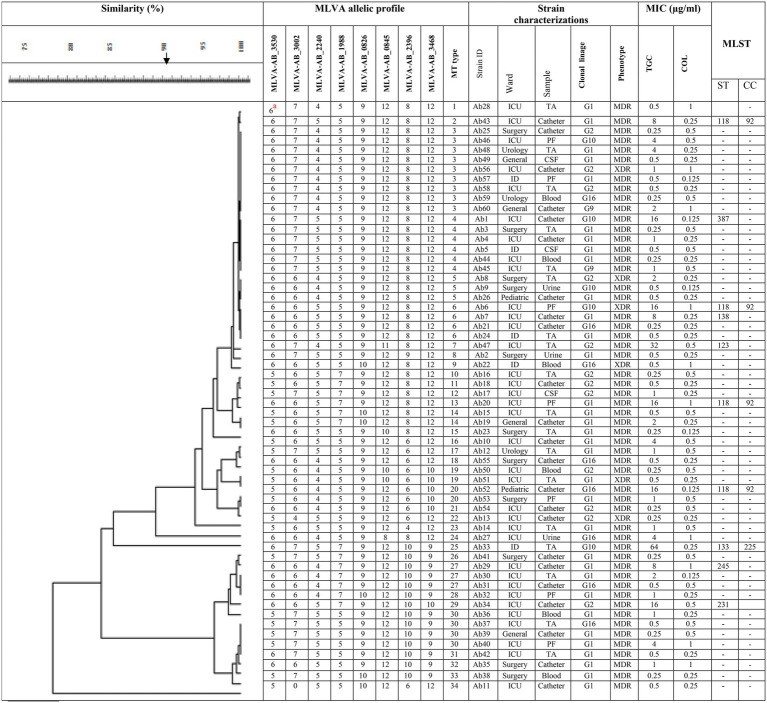
Dendogram shows the genetic diversity of 60 *A. baumannii* isolates by MLVA and MLST. Ab 1, *A. baumannii* number 1; TA, tracheal aspirates; PF, pleural fluid; IC,: International clonal linage; MDR, multidrug resistant; XDR, extensively drug-resistant; MIC, minimum inhibitory concentration; TGC, tigecycline; COL, colistin; CC, clonal complex; ST, sequence type. ^a^The number of repeats in VNTR alleles for each isolate.

International clone type analysis indicated five PCR-based groups (G1, G2, G9, G10, and G16) among *A. baumannii* isolates. Twelve (20%) and 33 (55%) isolates belonged to G2 (IC I) and G1 (IC II), respectively. Group 3 was not found in any of the isolates. As for other isolates, they belonged to 3 IC variants PCR-based groups, including 2 (3.3%), 5 (8.3%), and 8 (13.3%), and to G9, G10, and G16, respectively. The frequency of antibiotic resistance in three epidemic lineages is shown in Table 1. More than 55% of the isolates belonged to IC I and IC II were resistant to all the antibiotics, except for colistin and tigecycline. Overall, by the MLVA typing method, 60 *A. baumannii* isolates were grouped into 34 distinct MTs with 5 clusters and 25 singleton genotypes. The most isolates were in cluster 1(*n* = 19) and cluster 4 (*n* = 12), respectively. The most MTs were in cluster 4 (*n* = 7 MTs; Figure 1). In this study, the VNTR loci MLVA-AB_2,396 (Number of different repeats, *n* = 5), MLVA-AB_3,002 (*n* = 4) and MLVA-AB_0845 (*n* = 4) showed a high level of diversity (Figure 2). While most MTs had a single member, the three main MTs were MT30 (*n* = 4), MT6 (n = 4), MT4 (*n* = 6), and MT3 (*n* = 9), which together comprised over 35% of all isolates. MLST analysis for isolates with resistance to tigecycline revealed a total of seven different STs. Among these isolates, four isolates belonged to clonal complex 92 (CC 92; Ab 43, Ab 6, Ab 20, and Ab 52, corresponding to ST118). These isolates had a MIC range: 8–64 μg/ml and belonged to IC G1, G2, G10, and G16.

## Discussion

*A. baumannii* has become a main hospital pathogen, due to MDR strains and it is now considered one of the most important bacteria that causes hospital-acquired infections (HAIs), with mortality rate from 8 to 35% according to type of infection and strain as well as increase in health care expenditures and hospital stays (Antunes et al., 2014; Cornejo-Juárez et al., 2020). It is noteworthy that *A. baumannii* infection increases the mortality rate in the ICU (Playford et al., 2007; Heidary et al., 2017). *A. baumannii* is classified as an ESKAPE hospital pathogen due to its resistance to colistin and tigecycline. Since *A. baumannii* is an immediate threat to human health, a new antibiotic design is needed (Whiteway et al., 2022). The reason for antibiotic resistance is the acquisition of resistance genes through integrons from the environment or intra-species (Liu et al., 2014). Antibiotic resistance, high environmental stability, and failure to identify harmful toxins in the host and its genome indicate the potential for pathogenicity of *A. baumannii* based on “persist and resist” (Whiteway et al., 2022). If a patient is suspected of having antibiotic resistance, appropriate bacterial susceptibility testing should be performed, especially for patients admitted to the ICU. Imipenem susceptibility testing is necessary if the patient has previously used carbapenems and glycopeptides. In order to reduce the emergence and spread of PDR, continuous monitoring of *A. baumannii* resistance should be performed (Xie et al., 2018). In this study, multiplex PCR, MLVA, and MLST were performed to evaluate the clonality of *A. baumannii*. Also, the effectiveness of different antibiotics on hospital isolates was investigated. The results indicate that none of the strains were sensitive to the piperacillin/tazobactam and cephalosporins. There are different results for piperacillin/tazobactam resistance from around the world. Resistance is reported at 94% in the Poland (Grochowalska et al., 2017), and the sensitivity was 33.3% in India (Tewari et al., 2018).

Bacteria use different strategies to fight antibiotics (Robatjazi et al., 2021) such as the efflux pump that plays an important role in the emergence of multiple resistance to antibiotics especially in *A. baumannii* (Basatian-Tashkan et al., 2020). At present, MDR and XDR *Acinetobacter* have high frequency all over the world (Tafreshi et al., 2019). For example in a study that conducted in Iran, the frequency of MDR and XDR of *A. baumannii* was reported to be 94.2 and 71.2%, respectively (Maspi et al., 2016). In our study, the frequencies of MDR and XDR isolates were 90 and 10%, respectively. The difference between the results is probably due to geographical differences, the lack of a standard pattern in the definition of MDR and XDR (Magiorakos et al., 2012) and the different origin of the isolates. Over the past decade, carbapenem has been effective against *A. baumannii* and used as an alternative treatment for MDR infections (Ying et al., 2015). However, due to the high consumption of carbapenem, its resistance has increased (Ying et al., 2015). There are reports from all over the world of high resistance to carbapenems (Rynga et al., 2015; Nowak et al., 2017). The frequency of carbapenem-resistant isolates in the present study was 68.3%. In a systematic review meta-analysis that examined studies published in Iran between 1995 and 2017 on carbapenem-resistant isolates, the pooled frequency of carbapenem resistance was reported to be 85.1%. Among Iran’s neighbors, such as Afghanistan, Turkey, Pakistan, and Iraq, there was a high rate of carbapenem resistance in *A. baumannii* (Nasiri et al., 2020). The difference observed in the results of our study and the aforementioned study can be due to various reasons. The difference in the number of patients in the two studies can be one of these reasons. Another reason can be attributed to the fact that although all the studies included in the systematic review are from Iran, they are not from all parts and cities of Iran and perhaps for this reason, different percentages of the prevalence are seen in the two studies. In a report by El-Mahdy et al., 100% of *A. baumannii* isolates were resistant to cephalosporins (El-Mahdy et al., 2017). The results of the study by Safari and her colleagues in 2015 in Iran showed that the resistance to ceftazidime and cefotaxime was 95 and 100%, respectively (Safari et al., 2015). According to the results obtained from the present study, the similarity in the high resistance of *A. baumannii* to cephalosporin is determined in both studies.

Tigecycline is a new Glycycline that works very well against *A. baumannii*. Tigecycline was licensed first in the United States and later in Canada and the Middle East (Karmostaji et al., 2014). With its bacteriostatic activity, tigecycline is considered as the last line of treatment for MDR isolates (Zhang et al., 2022). Colistin and tigecycline are the main choices for treating CRAB, but there are concerns about their toxicity and effectiveness (Ying et al., 2015). In cases where colistin and imipenem-resistant *A. baumannii* are reported, tigecycline was used as a treatment option for the patient (Cai et al., 2012; Sarada et al., 2014). In 2006, all strains isolated in Iran were susceptible to tigecycline, but between 2011 and 2014, resistance to this antibiotic increased (Taheri et al., 2022). In a study conducted by Mohammadi Bardbari et al. in Iran, all clinical and environmental isolates of *A. baumannii* were sensitive to colistin and tigecycline (Mohammadi Bardbari et al., 2020). Rahimi et al. in Iran examined 80 isolates, 100% sensitive to colistin, and 6% resistant to tigecycline (Rahimi et al., 2018). The results of the present study confirm that tigecycline and colistin are still the most effective antibiotics, which is consistent with previously published reports (Salehi et al., 2018; Tewari et al., 2018; Boral et al., 2019). With the exception of a few studies that have reported high resistance of *A. baumannii* to tigecycline, the highest range of resistance to tigecycline in Iran has been reported between 2 and 38.4% (Razavi Nikoo et al., 2017). In a study by Morfin-Otero et al. on *A. baumannii* strains from around the world, a slight increase in MIC_90_ for tigecycline was shown to confirm the effectiveness of tigecycline remained more stable than that of other microbial agents between 2004 and 2009 (Morfin-Otero and Dowzicky, 2012). In a similar study by Mendes et al. on global *A. baumannii* spp., >90% of all *A. baumannii* strains and 95% of MDR *A. baumannii* were inhibited by tigecycline at ≤2 mg/L (Mendes et al., 2010).

In this study, 83.33% of all *A. baumannii* strains were inhibited at ≤2 μg/ml of tigecycline. According to the results obtained from the above studies and our study, it is considered that tigecycline can still be used as an effective antibiotic in the treatment of A. *baumannii* infections, but to prevent the occurrence of antibiotic resistance, the use of this antibiotic should be controlled and done carefully. Since *A. baumannii* has genomic flexibility and heterogeneous resistance, this facilitates the emergence of resistance to tigecycline and colistin.

The global population structure of *A. baumannii* is changing, so molecular typing is important. So far, different G variants have been responsible for infections acquired from the hospital in different areas, of which 5–24% of these variants have been resistant to tigecycline (Taheri et al., 2022). In previous studies in Iran, G_1_ (IC II) was the most dominant clonal lineage of *A. baumannii* (Hojabri et al., 2014; Taheri et al., 2022), which is consistent with our study. In the study of Taheri et al. (2022), conducted in Iran, it was found that the G variants rate had an increasing trend (4–43%) between 2006 and 2015. Also, the resistance to tigecycline from 2013 to 2015 had an increasing trend of 22–45% among the isolates of G variants. The results also showed an increase in XDR among these lineages. Therefore, the prevalence of antibiotic-resistant phenotypes could be a reason for the increase in G variants over time. The increase in tigecycline and XDR resistance in the G variants indicates that some of their components are shared (Taheri et al., 2022).

IC II is the most scattered lineage ever identified in at least 34 countries. IC I and IC II are also called global clones (GC; Gaiarsa et al., 2019). Initially, three high-resistance ICs were identified (IC1,2 and 3), which are responsible for the MLST IP/UO (Institute Pasteur/University of Oxford) scheme to CCs 1/109, 2/118, and 3/187 (Martins et al., 2013). In the present study, the results of multiplex PCR showed five PCR-based or IC lineage groups (G_1_, G_2_, G_9_, G_10,_ and G_16_), among which IC II and IC I were the most common. In the study conducted by Farshadzadeh et al., 8 IC types (G_1_, G_2_, G_6_, G_9_, G_10_, and G_15_–G_17_) were reported, the most common of which were IC II and IC I, respectively. They also found that IC variants had the highest resistance to tigecycline (Farshadzadeh et al., 2015). In a study conducted by Bahador et al. in Iran, IC-V was the most common, followed by IC II (Bahador et al., 2018), while in the study of Hojabri et al., IC II was the most common (Hojabri et al., 2014).

Also, in another study conducted in 2012 by Bahador et al. in Iran, IC II (58%) and IC I (29%) were the most common lineages (Bahador et al., 2015). Similarly, in a study conducted in Japan, the prevalence of IC II was higher than in non-IC II lineages (Matsui et al., 2018). *A. baumannii* belongs to IC I and IC II in connection with the high prevalence of hospitalization and virulence than others. Some of the surface features of these two lineages are different from each other, which may be a reason for the difference in their prevalence. For example, isolates belonging to IC II are immobile, do not form a pellicle, and have distinct polysaccharide capsules compared to IC I. IC I also has a very high hydrophobic property (Skerniškytė et al., 2019).

The basis of MLVA is determining the number of tandem repeat that are often located in the noncoding regions of the genome (Jenke et al., 2011). Multiple-locus variable-number tandem repeat analysis results indicate that isolates that are in the same G-type may be in different MLVAs or vice versa (Taheri et al., 2022). In studies in Iran (Najar Peerayeh and Karmostaji, 2015) and France (Hauck et al., 2012), due to partial deletion of locus, Abaum3468, Abaum3002, Abaum3530, and Abaum0826 had an amplification failure. In our study, the number of MTs was 34 different types, but in a similar study in Iran, 32 MTs were reported (Bahador et al., 2018). In the study of Azimi et al., there were five different molecular typing algorithms based on the size of VNTR (Azimi et al., 2016). In the present study, MLVA-AB-2396, MLVA-AB-3002, and MLVA-AB-0845 had the highest diversity, while in other studies conducted in Iran (Najar-Peerayeh and Karmostaji, 2019) and Spain (Villalón et al., 2015), MLVA-AB_0845 had the highest diversity. In another study conducted by Farshadzadeh and his colleagues in Iran, none of the CRAB *A. baumannii* isolates contained MLVA-AB_0845; instead, in their study, MLVA-AB_2,396 and MLVA-AB_3468 had the highest diversity (Farshadzadeh et al., 2015). In a study conducted by Karimi et al. in Iran, *A. baumannii* isolates were isolated from human and animal samples, and their typing pattern was determined using MLVA. Their research showed that 0845, 0826, and 3,406 VNTRs loci were present in all animal isolates and 3,406 VNTR loci were present in all human isolates (Karimi et al., 2021).

In a study conducted in Lithuania, it was found that there is no relationship between the specific years in which the study was conducted and the clustering of the isolates. On the other hand, it was found that most varieties belonged to Abaum_0845 and Abaum_0826 (Kirtikliene et al., 2021). A study conducted by Peerayeh et al. showed that different strains are circulating in two Iranian hospitals and the population of XDR *A. baumannii* in Iranian hospitals is genetically diverse, which necessitates preventive measures and strict control (Najar Peerayeh and Karmostaji, 2015). Multilocus sequence typing is a convenient way to describe bacterial populations and has the ability to cluster isolates into large clones (Villalón et al., 2015; Wareth et al., 2021). Therefore, with the reproducibility of this technique, the isolates can be compared globally with each other, and each can be placed directly in its CC (Rafei et al., 2014). In the present study, tigecycline-resistant isolates were placed in 7 different STs, 4 of which belong to CC92. In the study of Farshadzadeh and his colleagues, various STs from Iran were identified, among which ST118 was dominant (Farshadzadeh et al., 2015).

The results of various studies conducted in Iran have introduced CC92 as a common clone (Hojabri et al., 2014; Farshadzadeh et al., 2015) and has a global superiority (Ying et al., 2015). In a study conducted in China by Chang et al., CC92 was reported to be the most common CC resistant to carbapenem (Chang et al., 2015). Another study in China identified five different STs, and 85.2% of all clinical isolates belonged to CC92 (Ying et al., 2015). In a study conducted by Saffari et al. in Iran between 2014 and 2015, three main STs (ST848, ST451, and ST195) were identified, all of which belonged to CC92. None of the identified STs in Saffari’s study previously existed in Iran. However, the critical point is that ST195 was identified in the Arab countries of the Persian Gulf, so it seems that the emergence of this strain in Iran has occurred due to the transfer of microbial resistance from neighboring countries (Saffari et al., 2017). In another study conducted by Abhari et al. in Iran in 2017, the highest prevalence of clones belonged to ST10, followed by ST2, ST3, and ST513. ST10 was first identified in China and is highly virulent and associated with prevalence. The results of the above studies show that the prevalence of clones varies in each hospital and the geographical area (Abhari et al., 2019). There are several reasons for the prevalence of CC92, including high compatibility and survival, and resistance to antibiotic stress (Chang et al., 2015).

## Conclusion

Antibiotic resistance is an important issue of public health that threatens to undermine decades of medical progress. *A. baumannii,* one of the causes of nosocomial infections, is frequently resistant to carbapenem antibiotics, usually leaving colistin and tigecycline as the last-resort antibiotics. However, increasing tigecycline resistance and colistin’s nephrotoxicity severely restrict the use of these antibiotics. Finally, the results obtained from this study showed that the frequency of IC2 was much higher than other ICs, and considering the important role of this lineage in the occurrence of antibiotic resistance. Continuous investigation and tracking of different lineages to find resistance patterns is recommended. The diverse *A. baumannii* lineages in the current study highlight the need for designing an adequate surveillance program in each country based on the main circulating clone to select the appropriate therapy. Governments, researchers, and pharmaceutical companies should also be encouraged to design new antibiotics, given the growing resistance in *A. baumannii* and other pathogenic bacteria. On the other hand, controlling and monitoring the use of antibiotics in the treatment of *A. baumannii* infections plays an important role in controlling the prevalence of resistance.

## Data availability statement

The original contributions presented in the study are included in the article/Supplementary material, further inquiries can be directed to the corresponding authors.

## Ethics statement

The studies involving human participants were reviewed and approved by IR.MEDILAM.REC.1400.135. Written informed consent to participate in this study was provided by the participants’ legal guardian/next of kin.

## Author contributions

AM and SKh contributed to the study conception and design. Data collection and analysis were performed by SKh, BK, VK, SKa, NO, and MHas. The first draft of the manuscript was written by SKh, AM, MHad, and MK and all authors commented on previous versions of the manuscript. All authors contributed to the article and approved the submitted version.

## Conflict of interest

The authors declare that the research was conducted in the absence of any commercial or financial relationships that could be construed as a potential conflict of interest.

## Publisher’s note

All claims expressed in this article are solely those of the authors and do not necessarily represent those of their affiliated organizations, or those of the publisher, the editors and the reviewers. Any product that may be evaluated in this article, or claim that may be made by its manufacturer, is not guaranteed or endorsed by the publisher.

## Footnotes

^1^ http://pubmlst.org/abaumannii
