# Supplementary material for: Molecular typing and antibiotic resistance patterns among clinical isolates of Acinetobacter baumannii recovered from burn patients in Tehran, Iran
Source: Front Microbiol. 2022 Oct 21;13:994303. doi: 10.3389/fmicb.2022.994303 (PMC9664937; doi:10.3389/fmicb.2022.994303)
Supplement: Supplementary file 1 [file Table_1.DOCX]

Supplementary file1. PCR groups (G) based on the combination of amplicons in two multiplex PCR

| PCR group 2 | | | PCR group 1 | | | IC type | |
| --- | --- | --- | --- | --- | --- | --- | --- |
| *bla*_oxa-51-like_  162 bp | *ompA*  343 bp | *csuE*  580 bp | ompA  355 bp | *bla*_oxa-51-like_  559 bp | *csuE*  702 bp |  |  |
| - | - | - | + | + | + | IC II (G1) | |
| + | + | + | - | - | - | IC I (G2) | |
| - | + | - | - | + | + | IC III (G3) | |
| - | - | - | + | + | - | G4 | IC variants |
| - | - | - | + | - | - | G5 |  |
| + | + | - | - | - | + | G6 |  |
| - | - | + | + | - | - | G7 |  |
| - | - | + | + | + | - | G8 |  |
| + | - | + | + | - | - | G9 |  |
| - | + | + | - | + | - | G10 |  |
| + | + | - | - | - | - | G11 |  |
| - | - | - | - | + | - | G12 |  |
| - | - | - | + | - | + | G13 |  |
| - | - | - | - | + | + | G14 |  |
| + | - | - | - | - | + | G15 |  |
| - | - | + | - | + | - | G16 |  |
| + | - | - | + | - | + | G17 |  |
