# Supplementary material for: Molecular typing and antibiotic resistance patterns among clinical isolates of Acinetobacter baumannii recovered from burn patients in Tehran, Iran
Source: Front Microbiol. 2022 Oct 21;13:994303. doi: 10.3389/fmicb.2022.994303 (PMC9664937; doi:10.3389/fmicb.2022.994303)
Supplement: Supplementary file 2 [file Table_2.DOCX]

| **Primer name** | **Primer sequence (5-3)** | **Repeat size (bp)** | **Size of flanking regions (bp)** | **PCR condition** | | **Expected size (bp)** | **Reference** |
| --- | --- | --- | --- | --- | --- | --- | --- |
|  |  |  |  | **Annealing temp (^°^C)** | **Elongation time (S)** |  |  |
| MLVA-AB_3530_F | TGCAACCGGTATTCTAGGAAC | 60 | 121 | 55 | 45 | 462-522 | 26 |
| MLVA-AB_3530_R | CCTTGAACAACATCGATTACTGA |  |  |  |  |  |  |
| MLVA-AB _3002_F | GACTGAAGCAAGACTAAAACGT | 57 | 121 | 55 | 45 | 428 -482-539 | ʹʹ |
| MLVA-AB _3002_R | TCTGGGCAGCTTCTTCTTGAGC |  |  |  |  |  |  |
| MLVA-AB _2240_F | CCCGCAGTACATCATGGTTC | 99 | 494 | 55 | 60 | 624-723-826 | ʹʹ |
| MLVA-AB _2240_R | AGAACATGTATACGCAACTG |  |  |  |  |  |  |
| MLVA-AB _1988_F | GGCAAGGCATGCTCAAGGGCC | 26 | 77 | 55 | 45 | 195-213-291 | ʹʹ |
| MLVA-AB _1988_R | CAGTAGACTGCTGGTTAATGAG |  |  |  |  |  |  |
| MLVA-AB _0826_F | TGACTACTGAAACAGTTTTTG | 9 | 208 | 50 | 45 | 277-283-322-349-358-379 | ʹʹ |
| MLVA-AB _0826_R | ATGATTGTACCGAGTAAAAGA |  |  |  |  |  |  |
| MLVA-AB _0845_F | AATTTTAATTCCAAATTGCTCC | 7 | 105 | 50 | 45 | 119-175-189-217-238-308 | ʹʹ |
| MLVA-AB _0845_R | ACTTAAAATCGCATTTTTATCA |  |  |  |  |  |  |
| MLVA-AB _2396_F | CAAGTCCAATCAACTCATGATG | 6 | 105 | 55 | 45 | 147-159-201-219-225-231 | ʹʹ |
| MLVA-AB _2396_R | CTCCTGTAAGTGCTGTTCAGCC |  |  |  |  |  |  |
| MLVA-AB _3468_F | CAGAAGTCACTGCATCTGCAAC | 6 | 147 | 55 | 45 | 189-225-231 | ʹʹ |
| MLVA-AB _3468_R | CGGTTGAAATTTTTTATAATGAG |  |  |  |  |  |  |

Supplementary file 2. Primers, PCR conditions and characteristics of VNTRs used in this study
